# Alcohol Disrupts Human Liver Stem/Progenitor Cell Proliferation and Differentiation

**DOI:** 10.4172/2157-7633.1000205

**Published:** 2014-05-12

**Authors:** Xin Shi, Chia-Cheng Chang, Marc D Basson, Brad L Upham, Lixin Wei, Ping Zhang

**Affiliations:** 1 Department of Surgery, College of Human Medicine, Michigan State University, East Lansing, MI 48824, USA; 2 Department of Pediatrics and Human Development, College of Human Medicine, Michigan State University, East Lansing, MI 48824, USA; 3 Tumor Immunology and Gene Therapy Center, Eastern Hepatobiliary Surgery Hospital, The Second Military Medical University, Shanghai, China

**Keywords:** Alcohol, Liver, Stem cells, Proliferation, Differentiation, EGF, IL-6

## Abstract

**Objective:**

Excessive alcohol consumption injures the liver resulting in various liver diseases including liver cirrhosis. Advanced liver disease continues to be a major challenge to human health. Liver stem/progenitor cells (LSPCs) are tissue specific precursors with a distinct capacity of multi-lineage differentiation. These precursor cells may play an important role in the process of tissue injury repair and pathological transition of liver structures. At the present time, knowledge about the effect of alcohol on LSPC function during the development of alcoholic liver disease remains absent. This study was conducted to investigate changes in LSPC activity of proliferation and differentiation following alcohol exposure. The disruption of cell signaling mechanisms underlying alcohol-induced alteration of LSPC activities was also examined.

**Methods:**

Primary and immortalized human liver stem cells (HL1-1 cells and HL1-hT1 cells, respectively) were cultured in media optimized for cell proliferation and hepatocyte differentiation in the absence and presence of ethanol. Changes in cell morphology, proliferation and differentiation were determined. Functional disruption of cell signaling components following alcohol exposure was examined.

**Results:**

Ethanol exposure suppressed HL1-1 cell growth [as measured by cell 5-bromo-2-deoxyuridine (BrdU) incorporation] mediated by epidermal growth factor (EGF) or EGF plus interleukin-6 (IL-6) in an ethanol dose-dependent manner. Similarly, ethanol inhibited BrdU incorporation into HL1-hT1 cells. Cyclin D1 mRNA expression by HL1-hT1 cells was suppressed when cells were cultured with 50 and 100 mM ethanol. Ethanol exposure induced morphological change of HL1-1 cells toward a myofibroblast-like phenotype. Furthermore, ethanol down-regulated E-cadherin expression while increasing collagen I expression by HL1-1 cells. Ethanol also stimulated Snail transcriptional repressor (Snail) and α-smooth muscle actin (α-SMA) gene expression by HL1-1 cells.

**Conclusion:**

These results demonstrate that the direct effect of alcohol on LSPCs is inhibiting their proliferation and promoting mesenchymal transition during their differentiation. Alcohol interrupts LSPC differentiation through interfering Snail signaling.

## Introduction

Alcohol is the most frequently abused substance worldwide. Excessive alcohol consumption severely injures the liver, causing hepatitis, liver steatosis, fibrosis, and cirrhosis. Advanced liver disease continues to be a leading cause of death in the United States [1] and alcohol is responsible for up to 50% of deaths from chronic liver disease in Western countries [2–4]. Despite the enormous health and economic burden of alcoholic liver disease, little progress has been made in the treatment of this disease during the past half century. Lack of knowledge about mechanisms underlying the impairment of tissue injury repair process is a major hurdle obstructing improvement of patient care. Since alcohol causes severe metabolic disorder and functional derangement in hepatocytes, the role of hepatocyte proliferation in repairing alcoholic liver injury is restricted [2,5]. Residing in the biliary tree, particularly around the Canal of Hering, liver stem cells (LSCs) are ancestors of hepatic progenitors (oval cells) which can give rise to both hepatocytes and biliary epithelial cells [6]. Pathological examinations have observed that proliferation of liver stem/progenitor cells (LSPCs) is activated (ductular reaction) in patients with chronic liver diseases including those caused by excessive alcohol consumption [6–9]. The proliferative activation of LSPCs suggests that these precursors may play a significant role in the process of injury repair and tissue reconstruction in the diseased liver. In the past century, mechanisms underlying alcohol-induced injury to mature hepatocytes have been studied extensively. However, information about the effect of alcohol on LSPC homeostasis and activity during the development of alcoholic liver disease remains scant.

LSPCs are a small number of undifferentiated cells in the normal adult liver. They differ from mature hepatocytes and biliary epithelial cells in their extensive capacity for self-renewal and multi-lineage potential of differentiation. These undifferentiated ancestor cells typically do not share the same repertoire of surface receptor expression, signaling cascade organization, and metabolic pathway activity exhibited by fully differentiated parenchymal cells in the liver. The lineage commitment potential of liver precursor cells at various stages of differentiation can be distinctive. In addition, changes in the niche environment of LSPCs in the diseased liver may exert a profound influence on the fate of these precursors. Therefore, delineating the effects of alcohol on LSPC homeostasis and functional activity utilizing both the *in vivo* and *in vitro* model systems will be helpful for understanding the direct effect of alcohol and the indirect effect of alcohol-induced liver metabolic disorder and/or inflammation on LSPC function. Because most species of experimental animals are resistant to developing advanced alcoholic liver disease [2], no practical *in vivo* animal model is currently available for studying alcoholic liver disease. For the same reason, limited value exists for studying the effects of alcohol on LSPC function using *in vitro* cell culture models of liver precursor cells from animal origins. A few groups have tried to study precursor cell behavior in alcohol-related liver injury using human stem/progenitor cells of embryonic or hematopoietic origins [10,11]. Nevertheless, studies on extra-hepatic precursors may not provide definitive information. Our current study employed human LSC cell culture systems to characterize the alteration of liver precursor cell function following their exposure to alcohol. The focus of this investigation was to identify alcohol-induced defects of human LSPC proliferation and differentiation.

## Materials and Methods

### Culture of human LSPCs

**Figure 1:**
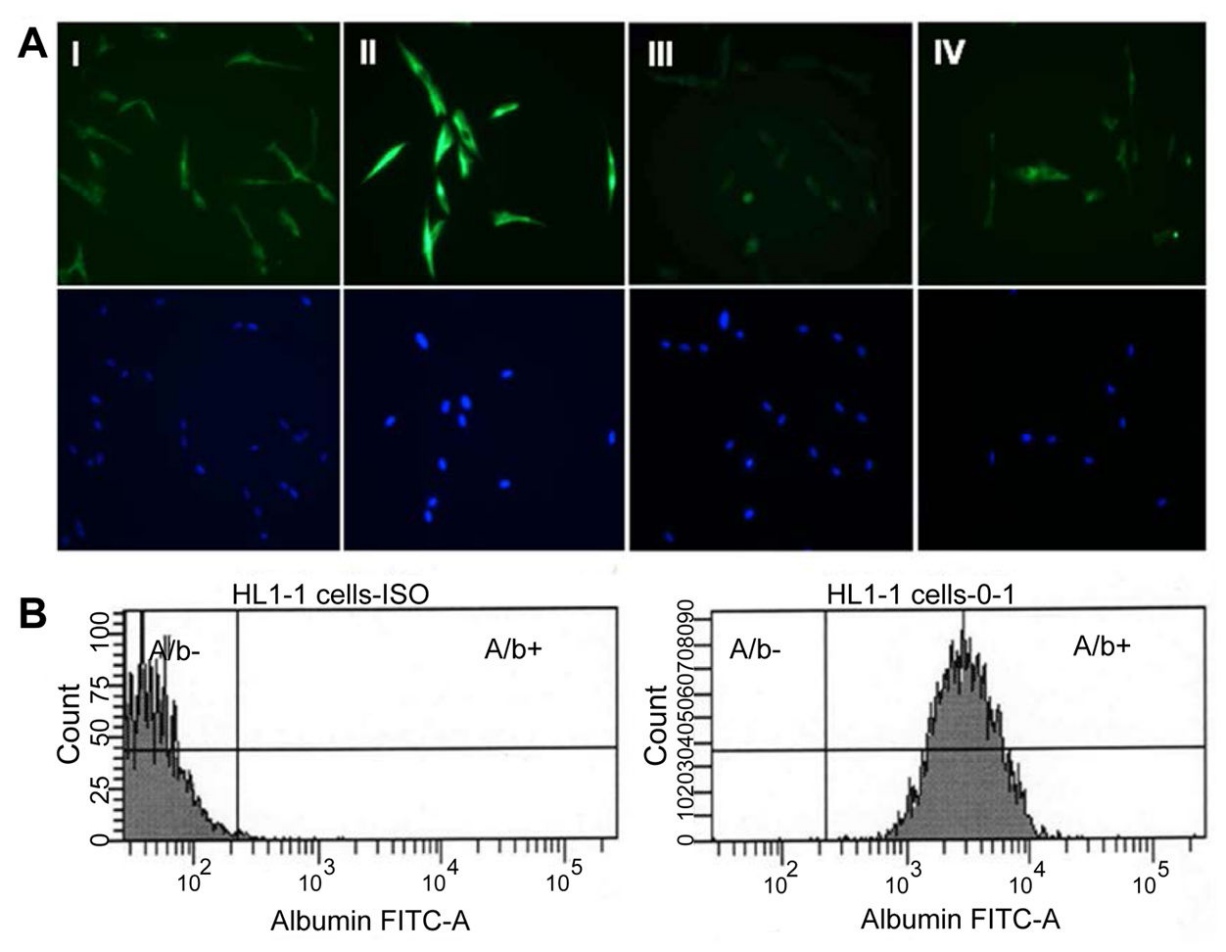
A: Expression of AFP (I), vimentin (II), Thy-1 (III), and cytokeratin 19 (IV) by HL1-1 cells. The lower panels are the corresponding nuclear staining with DAPI. B: Expression of albumin by HL1-1 cells cultured in hepatocyte differentiation medium for 2 weeks.

Our current investigation was conducted on cell culture models of HL1-1 cells. HL1-1 cells are human liver stem cells identified and characterized by Dr. Chang’s group [12,13]. These precursor cells were derived from a liver stem cell colony (HL1-1) in the culture of normal adult human liver cells. HL1-1 cells exhibit highly proliferative potential, express stem cell transcription factor (Oct-4) [14] and LSPC markers [α-fetoprotein (AFP), vimentin, thymocyte differentiation antigen 1 (Thy-1), and cytokeratin 19] (Figure 1), and have the ability to differentiate into albumin-producing cells (marker of hepatocytes) (Figure 1). In addition to this primary human liver stem cell model, a human telomerase reverse transcriptase (hTERT)-immortalized HL1- 1 cell line (HL1-hT1) has been developed by the same group through transfection of HL1-1 cells with pBABE-hygro-hTERT plasmids (from laboratory of Dr. Robert Weinberg). These human cell culture models are uniquely useful for studying toxicology and cell biology of human liver precursor cells [13,14].

For determining the effect of alcohol on LSC proliferation, primary and immortalized HL1-1 cells between 6 and 10 passages were cultured in proliferation medium [Keratinocyte-SFM medium (Life Technologies, Grand Island, NY) containing L-glutamine, recombinant human epidermal growth factor 1-53 (EGF 1–53) and bovine pituitary extract (BPE), 2 mM of N-acetyl-L-cysteine (Sigma- Aldrich Co. LLC. St. Louis, MO), 5 mM of nicotinamide (Sigma- Aldrich), 0.2 mM of ascorbic acid (Sigma-Aldrich), 10% fetal bovine serum (FBS, Life Technologies), and 1 μg/ml of gentamicin (Jackson Laboratories, Ltd., Amritsar)] without or with 50 ng/ml of recombinant human interleukin-6 (IL-6, Life Technologies) in 12- or 6-well tissue culture plates at 5×10^5^ cell or 3×10^6^ cells/well, respectively. Ethanol (Koptec, King of Prussia, PA) was added into the culture medium to establish different alcohol concentrations at 25, 50, and 100 mM. This range of alcohol concentrations is relevant to those frequently observed in blood samples from alcohol abusers. Control cells were cultured in the proliferation medium without ethanol. Cells were cultured at 37°C in incubators maintaining 5% CO_2_ and the corresponding alcohol environment [15]. Culture medium was changed every 48 h. 5-bromo- 2-deoxyuridine (BrdU, 1 μg/well, BD Biosciences, San Diego, CA) was added to the culture at either 48 or 4 h prior to termination of experiment.

For examining the effect of alcohol on LSC differentiation, primary HL1-1 cells between 6 and 10 passages were cultured in 6-well tissue culture plates precoated with collagen (50 μg/ml in PBS, calf skin type I collagen, Sigma-Aldrich) at 3×10^6^ cells/well in the proliferation medium for 3 days to achieve confluence. The cultures were then shifted to a differentiation medium [(Keratinocyte-SFM medium containing L-glutamine, EGF1-53 and BPE, 2 mM of N-acetyl-Lcysteine, 5 mM of nicotinamide, 0.2 mM of ascorbic acid, 20 ng/ml of recombinant human hepatocyte growth factor (HGF, R&D Systems, Inc., Minneapolis, MN), 1 mM sodium butyrate (Sigma-Aldrich), 20 ng/ml of dexamethasone (Sigma-Aldrich), 1% FBS, and 1 μg/ml of gentamicin]. Establishing different concentrations of ethanol in the cell culture system as well as culturing cells in incubators with 5% CO_2_ and the corresponding alcohol environment was the same as described above. Cell culture medium was changed every 72 h.

### Morphological examination

Phase contrast morphological examination of cultured cells was performed using the Olympus IX81 Imaging System with the PCA5 Slidebook Digital Image Acquisition Software.

#### Flow cytometry

At the end of cell culture, the culture medium was removed from each well. Attached cells in each well were washed with cold phosphate buffered saline (PBS, Sigma-Aldrich) and then removed by trypsin (1 ml/well, Life Technologies) digestion for 5 min. Cell suspension in each well was collected and mixed with 3 ml of cold culture media. After centrifugation at 500 g for 5 min at 4°C, the cell pellets were washed one time with cold PBS containing 1% bovine serum albumin (BSA, Sigma-Aldrich). For determination of E-cadherin expression, cells were fixed and permeabilized using a BD cytofix/ Cytoperm™ kit and the procedure provided by the manufacturer (BD Bioscience). Following fixation and permeabilization, cells were suspended in PBS containing 1% BSA and stained with FITC-conjugated mouse anti-human E-cadherin monoclonal antibody (1 μg antibody per 10^6^ cells, clone 36/E-Cadherin, recognizing the cytoplasmic domain of human E-Cadherin, BD Bioscience) or the isotype control antibody (clone eBM2a, BD Biosciences). After incubation for 15 min at room temperature in the dark, stained cells were washed with cold PBS containing 1% BSA. For determination of collagen I and albumin expression, cells were fixed and permeabilized using a BD cytofix/ Cytoperm™ kit and the procedure provided by the manufacturer (BD Bioscience). Following fixation and permeabilization, cells were suspended in PBS containing 1% BSA and stained with mouse anti-human collagen I monoclonal antibody (1 μg antibody per 10^6^ cells, clone COL-1, Sigma-Aldrich) and mouse anti-human albumin monoclonal antibody (1 μg antibody per 10^6^ cells, clone 188835, R&D Systems), respectively. After incubation for 15 min at room temperature in the dark, Alexa Fluor 488 conjugated goat anti-mouse IgG antibody (1 μg antibody per 10^6^ cells, Life Technologies) was added to each sample. The cell mixtures were further incubated for 15 min at room temperature in the dark. Cells for background staining were stained with Alexa Fluor 488 conjugated goat anti-mouse IgG antibody only. Stained cells were washed with cold PBS containing 1% BSA. For measuring BrdU incorporation, the cells were processed using a BD BrdU Flow Kit with the procedure provided by the manufacturer (BD Biosciences). At the end of the staining procedure, cells were suspended in 0.5 ml of PBS containing 1% paraformaldehyde. Analysis of cell E-cadherin, collagen I, and albumin expression as well as BrdU incorporation was performed on a FACSAria II cytometer with FACSDiva software (Becton Dickinson, San Jose, CA). A minimum of 5,000 cells were acquired in each sample. Average cell volume of the gated cell population was calculated using the equation of $V=4/3\pi D^{3}$, where D = mean channel of forward side scatter (MCFSC).

### Preparation of total RNA sample and quantitative real-time RT-PCR determination

Total RNA from cultured cells was isolated using the RNeasy^®^ Plus Mini Kit and procedures provided by the manufacturer (Qiagen, Valencia, CA). RNA (12 ng) was subjected to 2-step real-time reverse transcription and polymerase chain reaction (RT-PCR) using iScript™ Reverse Transcription Supermix kit and SsoFast™ EvaGreen^®^ Supermix kit (Bio-Rad, Hercules, CA), respectively, on the CFX96™ Real-Time System (Bio-Rad). The amplification primers used for determinations are listed as follows:

Human cyclin D1
Forward primer 5’-CATCTACACCGACAACTCCATC-3’
Reverse primer 5’-TCTGGCATTTTGGAGAGGAAG-3’
Human Snail
Forward primer 5’-GGAAGCCTAACTACAGCGAG-3’
Reverse primer 5’-CAGAGTCCCAGATGAGCATTG-3’
Human α-smooth muscle actin (α-SMA)
Forward primer 5’-AAT GCA GAA GGA GAT CAC GG -3’
Reverse primer 5’-TCC TGT TTG CTG ATC CAC ATC -3’
Human fibroblast-specific protein 1 (FSP1 or S100A4)
Forward primer 5’-AGT CAG AAC TAA AGG AGC TGC -3’
Reverse primer 5’-GAC ACA GTA CTC TTG GAA GTC C -3’
18S rRNA
Forward primer 5’-ATTGAAGTGAATCCCCAGACC-3’
Reverse primer 5’-TGAGCTTGTGTAAAAGTTGAACC-3’

These sets of primers for human cyclin D1, Snail, α-SMA, FSP1, and 18S rRNA were designed using Primer Express software (Life Technologies). The expression of cyclin D1, Snail, α-SMA, and FSP1 mRNA was determined by normalizing the cycle threshold (CT) number of their individual mRNA with that of 18S rRNA in each sample. Alcohol exposure induced changes of cyclin D1, Snail, α-SMA, and FSP1 mRNA expression are expressed as fold alterations over baseline expression by cells cultured simultaneously without exposure to alcohol.

### Statistical analysis

Data are presented as mean ± SEM. The sample size is indicated in each figure legend. Statistical analysis was conducted using one-way ANOVA followed by Student-Newman-Keuls test for comparisons among multiple groups. Mann-Whitney Rank Sum Test was performed for comparison between two groups. Statistical significance was accepted at *p*<0.05.

## Results

### HL1-1 cell growth and proliferation

**Figure 2:**
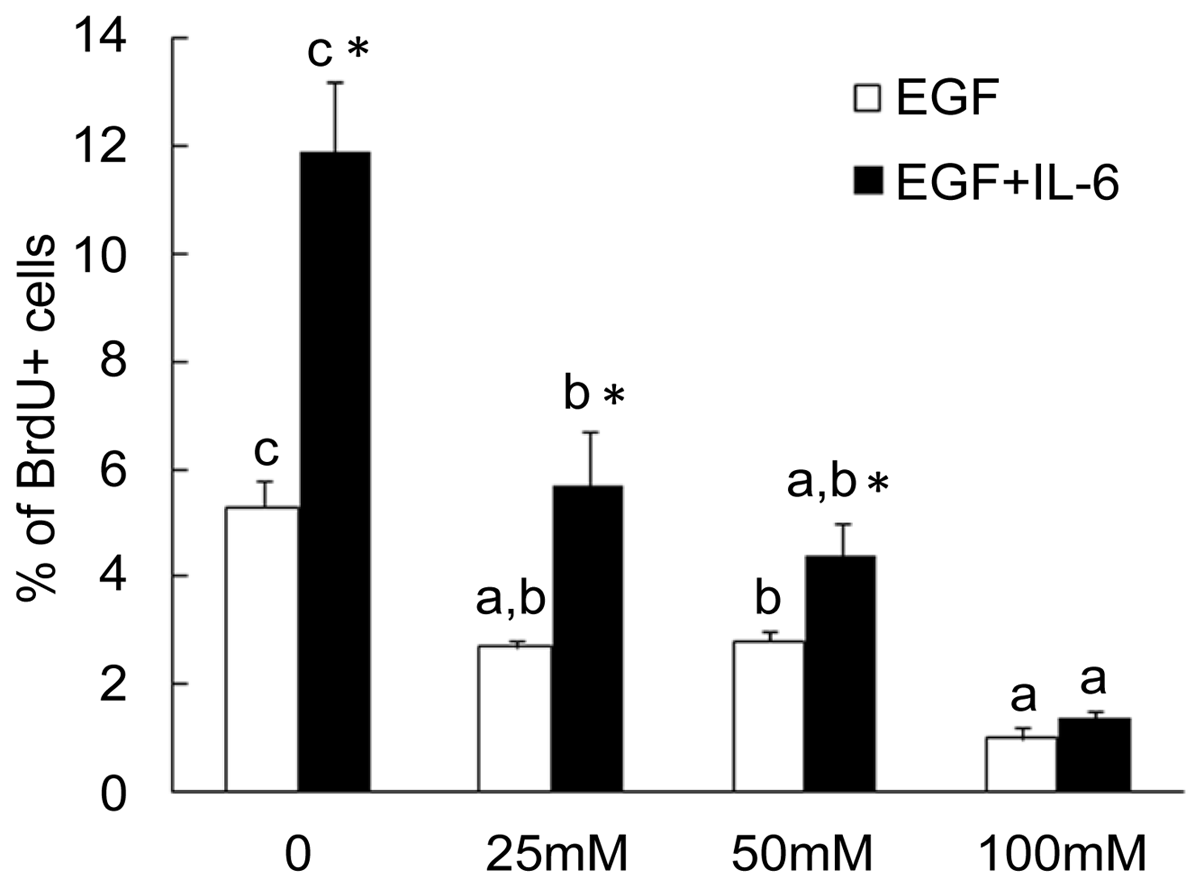
Alcohol exposure inhibited primary HL1-1 cell proliferation. HL1-1 cells were cultured in the proliferation medium with EGF or EGF+IL-6 for 72 h. BrdU was added into the culture medium during the last 48 h of cell culture. BrdU incorporation into cells was determined by flow cytometry. Values are mean ± SEM. N=4 sets of cell cultures. In each treatment, bars with different letters are statistically different (p<0.05); *: p<0.05 compared to cells cultured with EGF.

**Figure 3:**
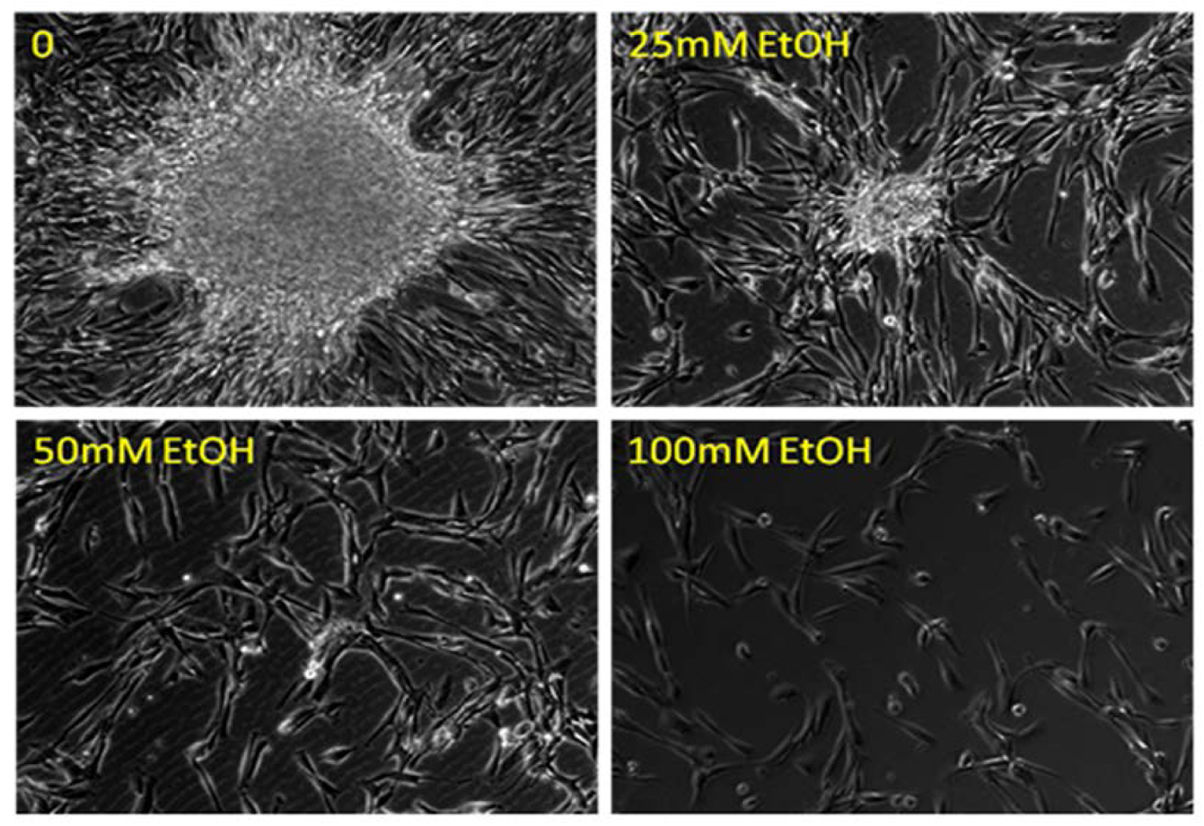
Representative images of immortalized HL1-hT1 cells cultured in the proliferation medium without and with different concentrations of alcohol for 7 days. The images captured on the Olympus IX81 Imaging System with 10X phase objective lens represent 4 sets of cell cultures.

**Figure 4:**
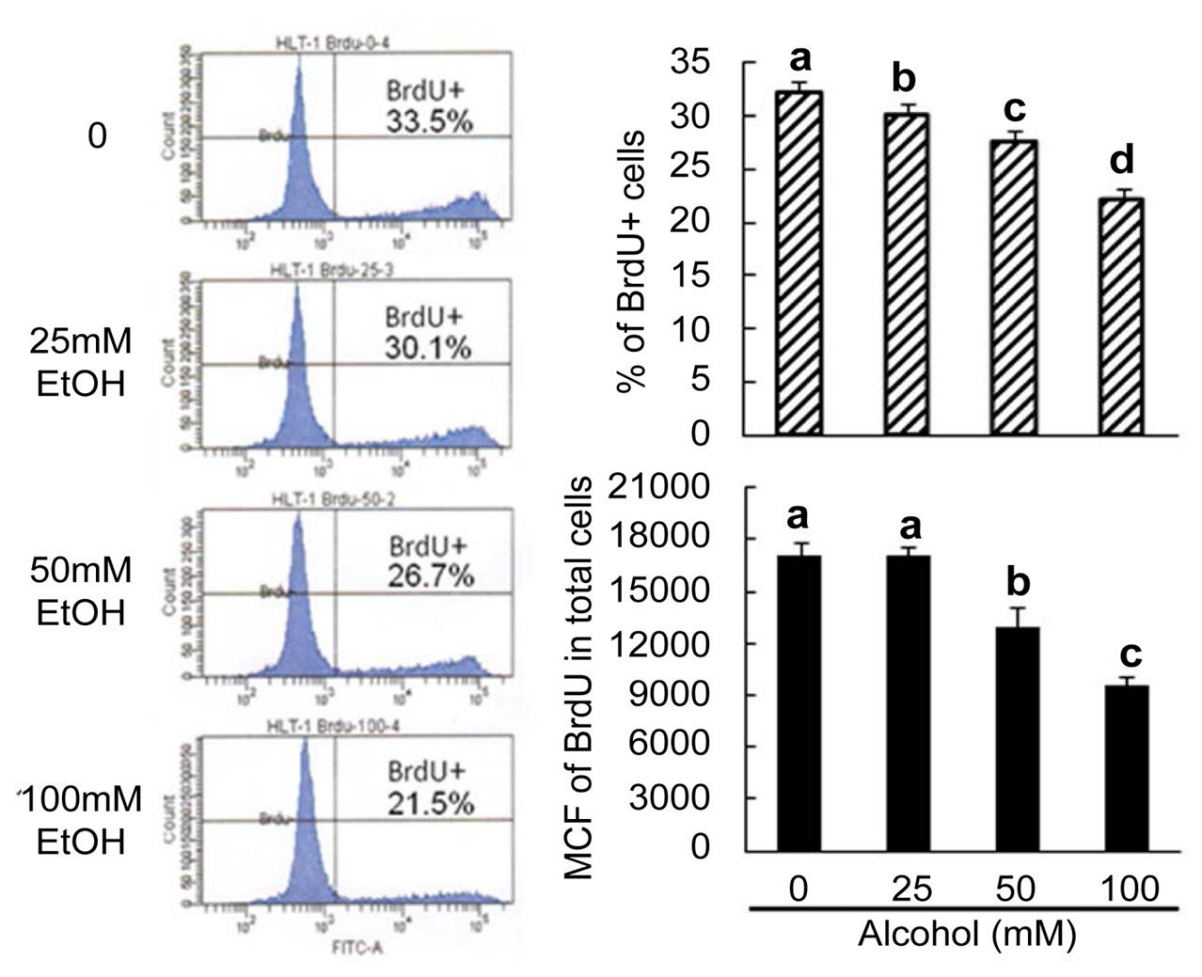
Changes in BrdU incorporation into immortalized HL1-hT1 cells. Cells were cultured in the proliferation medium without and with different concentrations of alcohol for 4 days. BrdU was added into the cell culture system during the last 4 h of culture. Values are mean ± SEM. N = 4 sets of cell cultures. MCF=mean channel fluorescence. Bars with different letters in each panel are statistically different (p<0.05).

EGF is a potent mitogen for liver precursor cells and hepatocytes [16,17]. During the development of alcoholic liver disease, inflammatory cytokine production in the liver is also significantly increased because of the inflammatory response [18]. Certain inflammatory cytokines, particularly IL-6, are potent stimuli for proliferation of liver precursor cells [19]. To evaluate the effect of alcohol on LSC growth and proliferation in response to EGF and IL-6, we initially employed the cell culture model of primary HL1-1 cells. These human LSCs grew steadily in proliferation medium containing EGF in our culture systems. BrdU is an analogue of thymidine that can be incorporated into DNA during DNA synthesis in proliferating cells. To quantify changes in primary HL1-1 cell self-renewal following alcohol exposure, we determined BrdU incorporation into primary HL1-1 cells during the last 48 h of a 3-day cell culture in the proliferation medium. As shown in Figure 2, primary HL1-1 cells grew well in the proliferation medium that contained recombinant human EGF 1-53. Addition of recombinant human IL-6 to the culture system significantly enhanced proliferation of primary HL1-1 cells as reflected by a marked increase in BrdU incorporation into these cultured cells. Exposure to alcohol resulted in inhibition of primary HL1-1 growth in an alcohol dose-dependent manner when cells were cultured in proliferation media containing either EGF alone or EGF plus IL-6. h-TERT-immortalized HL1-1 cells grew more vigorously in proliferation medium containing EGF, which facilitated analysis of cell proliferation. We therefore further characterized the direct effect of alcohol on the growth of HL1- hT1 cells. As shown in Figure 3, the growth of these precursor cells formed large sized colonies in the absence of alcohol. This pattern of cell growth is typically seen in culture systems of stem and/or upstream progenitors. Exposure to alcohol caused an alcohol dose-dependent inhibition of HL1-hT1 cell growth that was reflected by a reduction in cell colony size and a decrease in cell numbers in the culture system. Analysis of BrdU incorporation into HL1-hT1 cells during the last 4 h of a 4-day cell culture in the proliferation medium showed that approximately 1/3 (32.3 ± 0.5%) of HL1-hT1 cells incorporated with BrdU (BrdU^+^ cells) during this 4 h time window of cell culture in the absence of alcohol (Figure 4). Exposure of cells to alcohol resulted in decreases in the number of BrdU^+^ cells in the culture system in an alcohol dose-dependent manner. In addition, alcohol at concentrations of 50 and 100 mM caused a significant reduction of BrdU mean channel fluorescence (MCF, an index of BrdU incorporation extent; 24.3% and 44.0%, respectively) in the entire cell population of the culture system as compared to the BrdU MCF value in cells cultured without alcohol. These data confirm that alcohol exposure directly impairs LSPC activity of proliferation.

### Changes in cell volume and cyclin D1 mRNA expression

**Figure 5:**
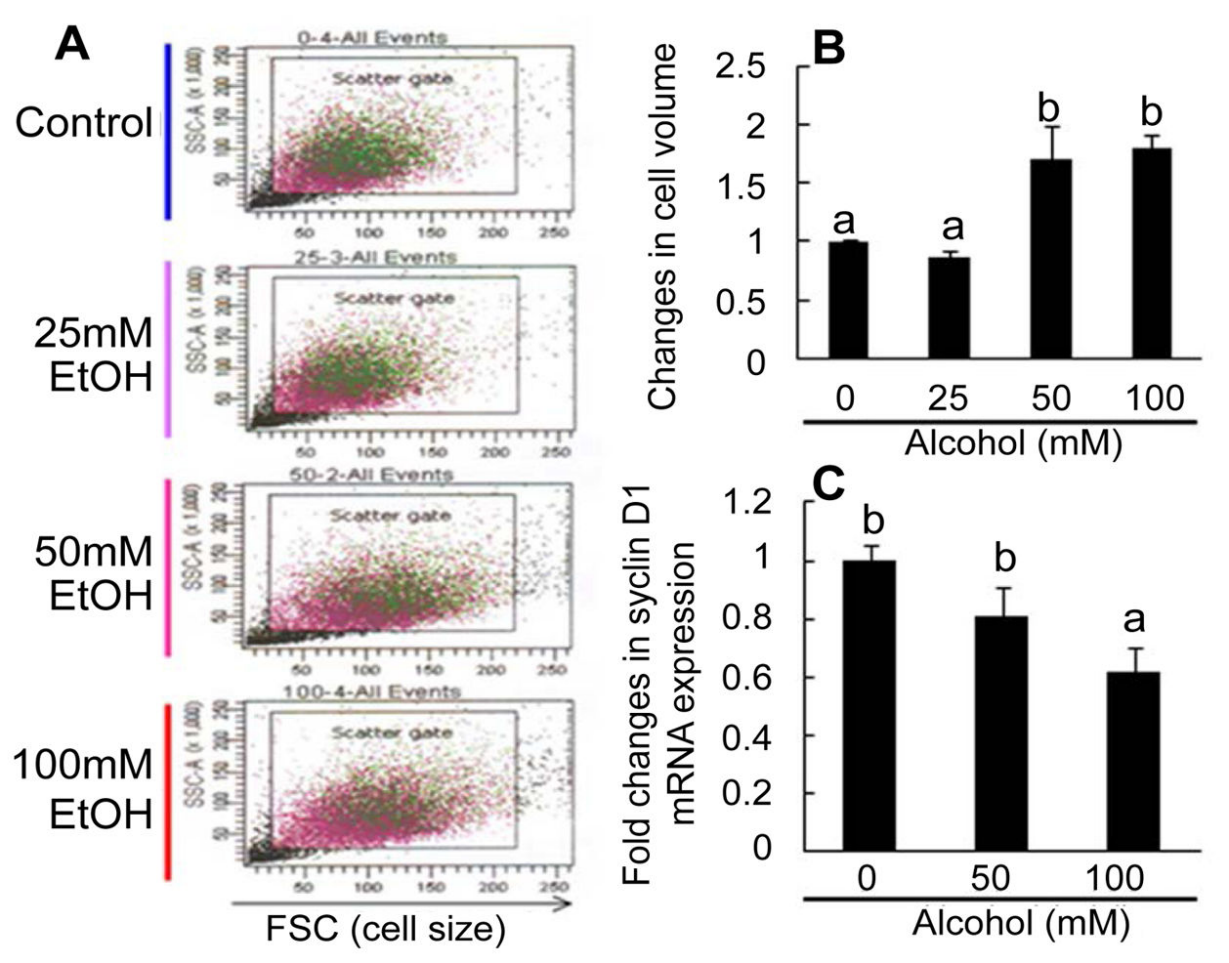
A and B: Changes in cell size of immortalized HL1-hT1 cells. Cells were cultured in the proliferation medium without and with different concentrations of alcohol for 4 days. Values are mean ± SEM. N = 4 sets of cell cultures in each group. Bars with different letters in panel B are statistically different (p<0.05). C: Changes in cyclin D1 expression by immortalized HL1- hT1 cells. Cells were cultured in the proliferation medium without and with different concentrations of alcohol for 24 h. Values are mean ± SEM. N = 5 sets of cell cultures. FSC=forward side scatter. Bars with different letters are statistically different (p<0.05).

In flow cytometric dot plots of cell scatters, the values of the forward side scatter (FSC, representing cell size) in the gated population of HL1-hT1 cells cultured with alcohol at 50 and 100 mM were markedly increased (Figures 5A and 5B). This increase in the size (or volume) of HL1-hT1 cells occurred concomitantly with the reduction in cell activity of proliferation (as reflected by BrdU incorporation in these cells shown in Figure 4) during culture with alcohol.

EGF-stimulated liver precursor cell proliferation involves cell signaling through activation of p44/42 mitogen-activated protein kinase [p44/42 MAPK or extracellular-signal-regulated kinases (ERK)]-cyclin D cascade. Cyclin D1 is an important member of the D-type cyclin family, which is commonly regulated at the transcriptional level in this signaling system. Our results showed that alcohol exposure caused a rapid down-regulation of cyclin D1 mRNA expression by HL1-hT1 cells cultured in proliferation medium (Figure 5C). The level of cyclin D1 mRNA expression by cells cultured with 100 mM alcohol decreased to 61.7% of the value seen in cells cultured without alcohol.

### Change in morphology during HL1-1 cell differentiation

**Figure 6:**
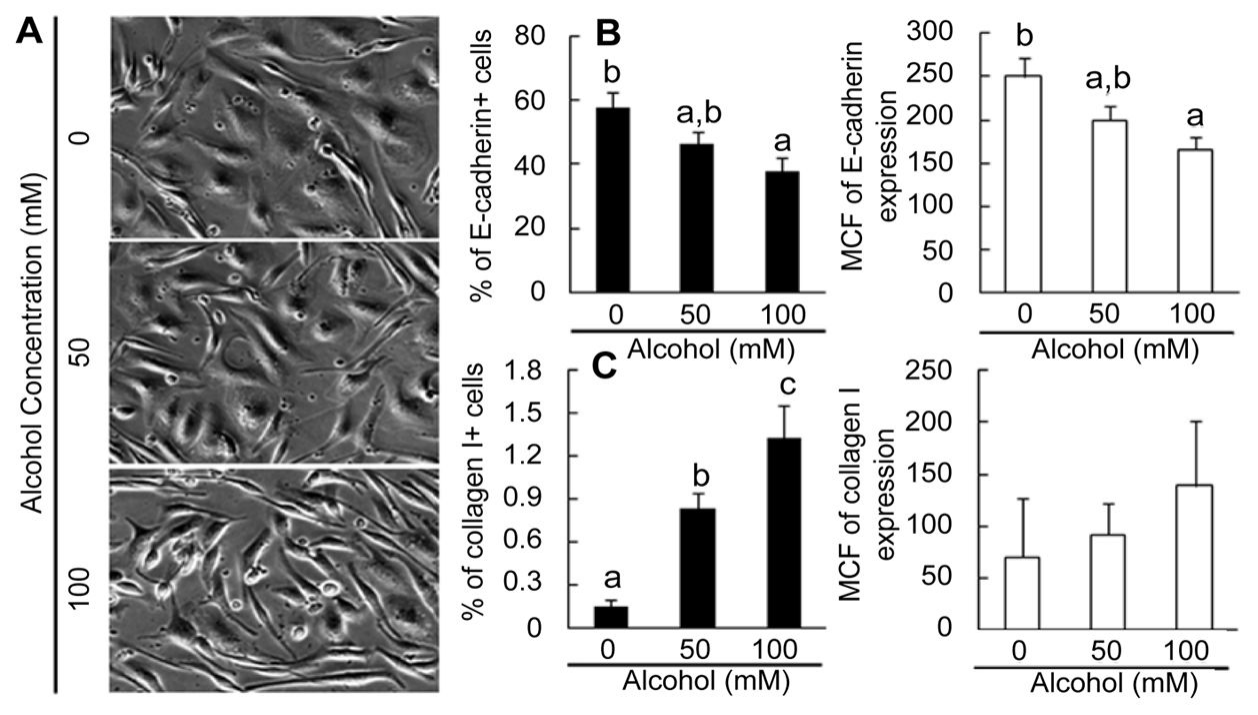
A: Morphological changes of primary HL-1-1 cells. Cells were cultured in the differentiation medium without and with different concentrations of alcohol for 10 days. The images captured on the Olympus IX81 Imaging System with 10X phase objective lens represent 5 sets of cell cultures. B: Changes in E-cadherin expression by primary HL1-1 cells. Cells were cultured in the differentiation medium without and with different concentrations of alcohol for 2 weeks. N=4 sets of cell cultures. C: Changes in collagen I expression by primary HL1-1 cells. Cells were cultured in the differentiation medium without and with different concentrations of alcohol for 2 weeks. N=4 sets of cell cultures. Values are mean±SEM. MCF=mean channel fluorescence. Bars with different letters in each panel are statistically different (p<0.05).

Primary HL1-1 cells cultured in differentiation medium in type I collagen coated plates for 10 days developed a typical hepatocyte-like epithelial morphology (Figure 6A). While increasing in cell size, they spread and formed flattened, nearly contiguous cell monolayers. Exposure to 50 mM alcohol caused a heterogeneous change of cell shapes. Many cells started forming projections from their cell bodies. This heterogeneous change of cell morphology became much more prominent in cells cultured with 100 mM alcohol. In comparison to cells cultured in differentiation medium without alcohol, primary HL1- 1 cells in cultures with 100 mM alcohol failed to develop the normal morphology characteristic of hepatocyte-like epithelial differentiation. Instead, the majority of cells maintained the less differentiated spindle-like shape. A large number of cells formed long and thin projections resembling those typically seen in cultures of myofibroblasts.

### Changes in E-cadherin and collagen I expression

E-cadherin, an important member of the cadherin superfamily, is expressed by cells of epithelial origin. E-cadherin plays a critical role in maintaining hepatocyte adherence and survival [20,21]. E-cadherin expression increases in association with LSPC differentiation [22]. Therefore, we examined E-cadherin expression by primary HL1- 1 cells cultured in differentiation medium. At the end of 2 weeks of culture, cells exposed to alcohol (50 and 100 mM) during the culture showed a marked reduction of E-cadherin expression (Figure 6B). The alcohol-induced inhibition of E-cadherin expression was consistent with the morphological change of primary HL1-1 cells cultured in differentiation medium containing alcohol. Furthermore, this reduction in E-cadherin expression was associated with a concomitant increase in collagen I expression by primary HL1-1 cells cultured in differentiation medium containing alcohol (Figure 6C). These contrasting alterations in E-cadherin and collagen I expression further suggest that exposure to alcohol impairs cell differentiation toward a hepatocyte phenotype.

### Changes in Snail, α-SMA, and FSP1 mRNA expression

**Figure 7:**
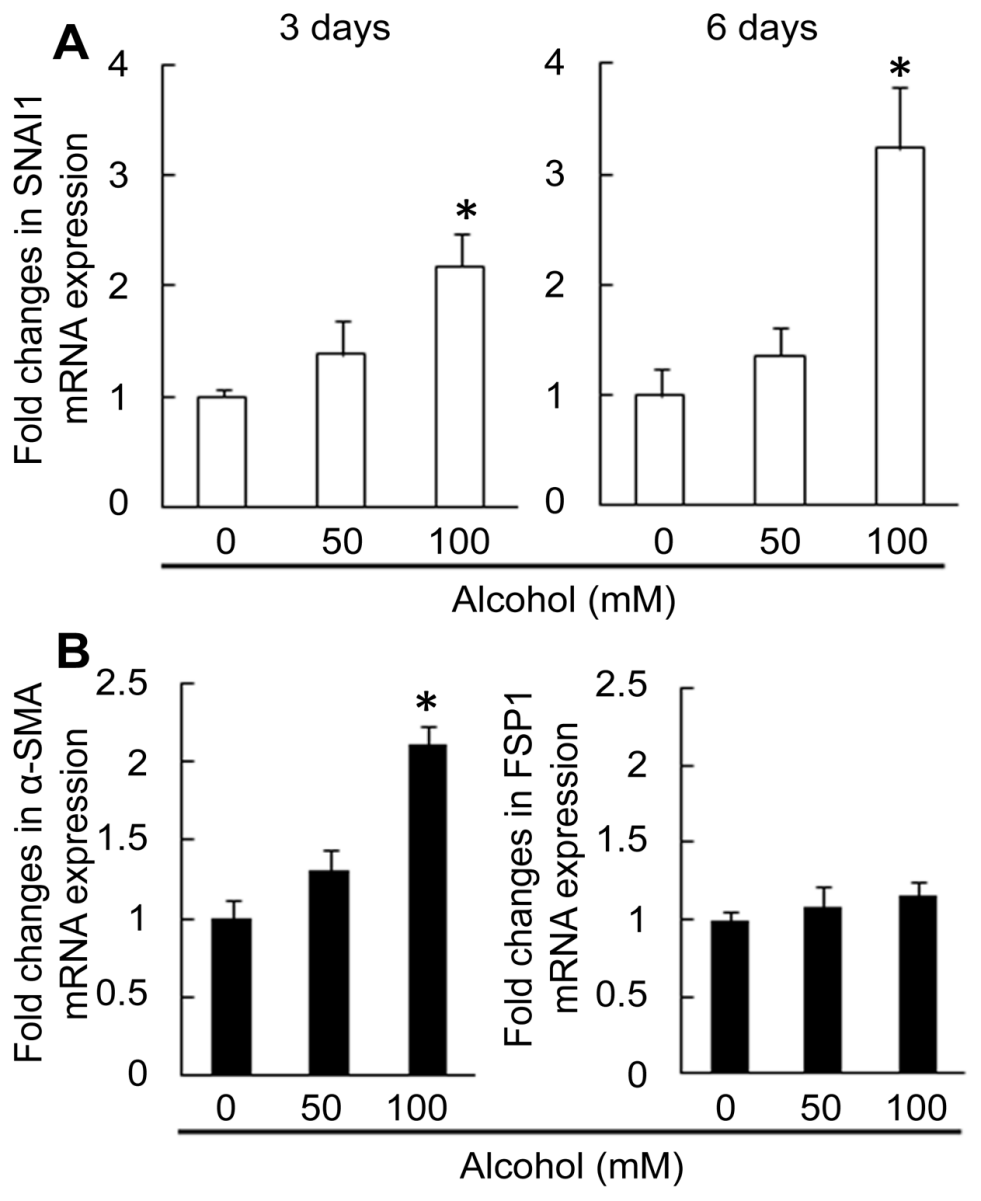
A: Changes in Snail mRNA expression by primary HL1-1 cells. Cells were cultured in the differentiation medium without and with different concentrations of alcohol for 3 and 6 days. N=5-6 sets of cell cultures. *: p<0.05 compared to other groups. B: Changes in α-SMA and FSP1 mRNA expression by primary HL1-1 cells. Cells were cultured in the differentiation medium without and with different concentrations of alcohol for 6 days. N=6 sets of cell cultures. *: p<0.05 compared to other groups.

The zinc finger protein Snail has been reported to repress E-cadherin expression [23]. We determined Snail mRNA expression by primary HL1-1 cells cultured in differentiation medium. As shown in Figure 7A, alcohol exposure caused a significant increase in Snail mRNA expression by primary HL1-1 cells in the differentiation culture system. These data suggest that Snail signaling may be involved in mediating the inhibition of E-cadherin expression by cultured primary HL1-1 cells following exposure to alcohol and possibly epithelial to mesenchymal transition (EMT) during alcohol-induced disruption of LSPC differentiation. Alpha-SMA is a cardinal marker of myofibroblasts in the liver [24,25], while FSP1 is a maker for inflammatory macrophages from the extrahepatic origin [26]. Alcohol exposure also resulted in a significant up-regulation of α-SMA mRNA expression by primary HL1-1 cells in the differentiation culture system (Figure 7B). However, FSP1 mRNA expression by these cells was not affected by alcohol in the differentiation culture system.

## Discussion

Pathological examinations of patients with alcoholic liver disease have repeatedly shown the activation of LSPC proliferation [27–29]. In particular, the extent of LSPC proliferation or ductular reaction has been found to correlate with the severity of alcoholic liver disease [28,29]. Extensive proliferation of LSPCs predicts the increase in short-term mortality in patients with alcoholic hepatitis, an acute event in chronic alcoholic liver disease that develops in up to 20% of patients with heavy consumption of alcohol [29]. Experimental studies on a murine model of alcoholic liver injury have revealed that increased oxidative stress in the liver inhibits the proliferation of mature hepatocytes, which is associated with the ductular reaction in the liver [27]. In animals with the greatest oxidative stress, mature hepatocyte proliferation is inhibited most, and the greatest number of oval cells accumulates. At the present time, it remains unclear how in vivo alcohol exposure exerts contrasting effects on the growth of liver cells at different stages of differentiation, i.e., inhibiting proliferation of mature hepatocytes while promoting the expansion of immature LSPC population in the liver.

The influences of alcohol consumption on liver cell metabolism and function are complex, including the primary effect of toxicity caused by alcohol and its derived metabolites as well as the secondary effect of the inflammatory response. Cell types at a given stage of differentiation can be sensitive to injuries caused by certain factors while relatively resistant to those induced by the others. Furthermore, the final fate of a specific cell type in the liver of alcoholic hosts is ultimately determined by the combined effects of involved factors. Among factors contributing to LSPC proliferation, EGF, a low-molecular-weight polypeptide of approximately 6 kDa, is a potent mitogen for liver precursor cells and hepatocytes [16,17]. Studies have shown that EGF levels in the systemic circulation are stable or even increased in individuals consuming alcohol [30,31]. Both EGF mRNA and protein levels are highly elevated in cirrhotic liver tissues [32]. The increase in EGF expression is particularly localized to regenerative hepatic nodules and bile duct epithelia of the cirrhotic liver. During the development of alcoholic liver disease, hepatic production of proinflammatory cytokines including IL-6, tumor necrosis factor (TNF)-α, and TNF-like weak inducer of apoptosis (TWEAK) is significantly increased [18,33,34]. These mediators are also involved in promoting LSPC proliferation. In order to define the direct effect of alcohol on LSPC proliferation in response to these mitogenic stimuli, we monitored the growth of primary HL1- 1 cells cultured in proliferation medium containing EGF or EGF plus IL-6. EGF maintained a stable proliferation of cultured primary HL1-1 cells. Addition of IL-6 to the culture system significantly augmented cell proliferation. An interesting observation is that alcohol exposure caused inhibition of primary HL1-1 cell proliferation in response to EGF or EGF plus IL-6 in an alcohol dose-dependent manner. We further characterized the inhibitory effect of alcohol on human LSC proliferation in the culture system of immortalized HL1-hT1 cells. Our results showed that alcohol exposure suppressed BrdU incorporation into these cells during their culture in proliferation medium containing EGF, which was associated with an increase in cell size and volume. These data indicate that the direct effect of alcohol on human LSPC proliferation in response to mitogen stimuli is inhibitory rather than stimulatory. The observed activation of LSPC proliferation or ductular reaction by pathological examinations of patients with alcoholic liver disease may be mediated through other associated factors instead of the direct effect of alcohol on LSPC growth. During the development of alcoholic liver disease, production of inflammatory mediators including growth factors and cytokines in the liver is significantly increased. It is most likely that the stimulatory effect of growth factors and cytokines produced in the liver during the inflammatory response may override the inhibitory effect of alcohol on LSPC proliferation, resulting in the overall increase in LSPC proliferation and ductular reaction in the liver. Therefore, extensive proliferation of LSPCs in alcohol liver disease may correlate with the severity of inflammation and tissue injury in the diseased liver.

EGF stimulates growth of the targeted cells through activation of cell surface EGF receptors (EGFR). Binding of EGF to EGFR activates p44/42 MAPK (or ERK1/2) through the Ras-Raf- ERK kinase (MEK) 1/2 cascade [35,36]. Activation of p44/42 induces expression of cyclin D and therefore enhances cyclin D-cyclin-dependent kinase 4/6 (CDK4/6) activity, which promotes cell cycling [37]. Engagement of IL-6 and IL-6R also promote cyclin D-mediated cell proliferation through activation of the signal transducer and activator of transcription 3 (STAT3) pathways [38–40]. Results of our current study demonstrated that alcohol exposure suppressed cyclin D1 expression by immortalized HL1-1 cells in the culture system. These data indicate that alcohol impairs EGFR signaling in mediating LSPC proliferation. Previous investigations from our group have shown that alcohol inhibits p44/42 activation in extra-hepatic stem/progenitor cells [41]. Studies by others have reported that alcohol impairs EGFR function in hepatocytes of rodent origin [42]. Further effort in delineating alcohol-induced disruption of EGFR and IL-6R signaling system will improve understanding mechanisms underlying the inhibitory effect of alcohol on EGF- and IL-6-stimulated LSPC proliferation.

In order to effectively repair tissue injury, the liver needs to secure a sufficient pool of precursor cells through maintaining and/ or enhancing their proliferation. Furthermore, these precursors should be able to differentiate into fully functional parenchymal cells. However, pathological examinations have shown that the size of the entire liver typically decreases along with a significant reduction of parenchymal hepatocyte mass while increasing in fibrous septa during the development of alcoholic liver disease, particularly to the stage of alcoholic liver cirrhosis [4]. In patients with alcoholic liver disease, strong activation of LSPC proliferation is commonly associated with severe liver injury and worse outcomes [28,29].

These pathological and clinical features suggest that defects of liver precursor cell function exist in the process of repairing alcoholic liver injury. Extensive LSPC proliferation or ductular reaction associated with alcoholic liver disease appears not effectively contributing to the repair of liver parenchyma or the restoration of the lost hepatocyte population. Instead, these proliferating precursors may promote the development of fibrosis and cirrhosis. Studies have shown that in certain pathological circumstances, LSPC may trans-differentiate into mesenchymal cell types exhibiting fibroblastic features through epithelial-mesenchyrmal transition (EMT) [43,44]. In our current study, primary HL1-1 cells cultured for 10 days in differentiation medium containing recombinant human HGF showed a morphological change toward typical hepatocyte-like epithelial differentiation. Exposure to alcohol disrupted this normal differentiation process. Most cultured HL1-1 cells maintained a spindle-like shape and started to form long and thin projections. These morphological changes are similar to those seen in myofibroblasts. Hepatic myofibroblasts are major source of fibrillar and nonfibrillar matrix components in the development of liver fibrosis and cirrhosis [45,46]. Although stellate cell activation/transformation serves as an important source for myofibroblasts [47–49], recent studies have revealed that EMT in liver cell types of epithelial origin contributes to the accumulation of hepatic myofibroblasts [45,50].

E-cadherin is a general marker of epithelial cells. In the liver, hepatocytes, biliary epithelial cells, and LSPCs express E-cadherin [51]. Cell expression of E-cadherin is down-regulated during EMT [23,52]. In parallel with the change of morphology, primary HL1-1 cells cultured in the differentiation medium containing alcohol showed a down-regulation of E-cadherin expression. Concomitantly, collagen I expression by these cells was up-regulated. The Snail transcriptional repressor is a key EMT regulator [53]. Snail binds to the proximal promoter region of the CDH1 gene for the EMT induction through E-cadherin repression [23]. In association with the down-regulation of E-cadherin expression, alcohol exposure significantly up-regulated Snail mRNA expression by primary HL1-1 cells in our differentiation culture system. These data suggest that alcohol-induced disruption of LSPC differentiation and promotion of EMT most likely involve activation of Snail signaling. Along with the apparently switched track of cell differentiation, α-SMA {the cardinal marker of myofibroblasts in the liver [24,25]} mRNA expression by primary HL1-1 cells was also significantly up-regulated following their exposure to alcohol. Interestingly, gene expression of FSP1 {a maker for inflammatory cells from the extrahepatic orgin [26]} by primary HL1-1 cells was not affected by alcohol exposure during cell culture under the differentiation condition. These different patterns of change in cell marker gene expression are in consistent with the specific liver origin of HL1-1 cells. Notch signaling mediates Snail expression [54]. Alcohol exposure has been reported to activate Notch signaling in human endothelial cells [55]. In addition, studies have shown activation of Hedgehog signaling in the liver of alcoholic hosts [28]. Hedgehog signaling may also remotely promote expression of snail through inducing activation of Notch signaling [54].

Our *in vitro* models of HL1-1 and HL1-hT1 cells allow assessing the direct effect of alcohol on LSPC proliferation and differentiation in response to appropriate stimuli under the conventional culture condition. However, these culture models do have limits in representing the in vivo status of LSPCs residing in their natural niche environment. Particularly, the oxygen tension in human liver tissue is commonly lower than that in the ambient atmosphere. Further characterization of precursor cell properties in the culture system with an appropriately low oxygen environment and comparison of their functional/metabolic alterations following exposure to alcohol will provide a deeper insight into the disruption of LSPC activity in alcohol abusers. Effort in this respect will also facilitate identifying critical targets for developing therapeutic intervention to treat liver injury caused by excessive alcohol consumption.

## References

[1] Murphy SL, Xu J, Kochanek KD (2012). Deaths: Preliminary Data for 2010. National Vital Statistics Reports.

[2] Gao B, Bataller R (2011). Alcoholic liver disease: pathogenesis and new therapeutic targets. Gastroenterology.

[3] Orholm M, Sørensen TI, Bentsen K, Høybye G, Eghøje K (1985). Mortality of alcohol abusing men prospectively assessed in relation to history of abuse and degree of liver injury. Liver.

[4] Arteel G, Marsano L, Mendez C, Bentley F, McClain CJ (2003). Advances in alcoholic liver disease. Best Pract Res Clin Gastroenterol.

[5] Saso K, Moehren G, Higashi K, Hoek JB (1997). Differential inhibition of epidermal growth factor signaling pathways in rat hepatocytes by long-term ethanol treatment. Gastroenterology.

[6] Duncan AW, Dorrell C, Grompe M (2009). Stem cells and liver regeneration. Gastroenterology.

[7] Van Eyken PK, Vos RD, Desmet VJ, Hall P (1995). Progenitor (‘‘stem’’) cells in alcoholic liver disease?. Alcoholic Liver Disease.

[8] Feng D, Kong X, Weng H, Park O, Wang H (2012). Interleukin-22 promotes proliferation of liver stem/progenitor cells in mice and patients with chronic hepatitis B virus infection. Gastroenterology.

[9] Tan J, Hytiroglou P, Wieczorek R, Park YN, Thung SN (2002). Immunohistochemical evidence for hepatic progenitor cells in liver diseases. Liver.

[10] Pal R, Mamidi MK, Das AK, Gupta PK, Bhonde R (2012). A simple and economical route to generate functional hepatocyte-like cells from hESCs and their application in evaluating alcohol induced liver damage. J Cell Biochem.

[11] Piscaglia AC, Zocco MA, Di Campli C, Sparano L, Rutella S (2005). How does human stem cell therapy influence gene expression after liver injury? Microarray evaluation on a rat model. Dig Liver Dis.

[12] Chang CC, Tsai JL, Kuo KK, Wang KH, Chiang CH (2004). Expression of Oct-4, alpha fetoprotein and vimentin and lack of gap-junctional intercellular communication (GJIC) as common phenotypes for human adult liver stem cells and hepatoma cells. Proc Am Assoc Cancer Res.

[13] Kim S, Dere E, Burgoon LD, Chang CC, Zacharewski TR (2009). Comparative analysis of AhR-mediated TCDD-elicited gene expression in human liver adult stem cells. Toxicol Sci.

[14] Tai MH, Chang CC, Kiupel M, Webster JD, Olson LK (2005). Oct4 expression in adult human stem cells: evidence in support of the stem cell theory of carcinogenesis. Carcinogensis.

[15] Zhang Z, Bagby GJ, Stoltz D, Oliver P, Schwarzenberger PO (2001). Prolonged ethanol treatment enhances lipopolysaccharide/phorbol myristate acetate-induced tumor necrosis factor-alpha production in human monocytic cells. Alcohol Clin Exp Res.

[16] Isfort RJ, Cody DB, Stuard SB, Randall CJ, Liller C (1997). The combination of epidermal growth factor and transforming growth factor-beta induces novel phenotypic changes in mouse liver stem cell lines. J Cell Sci.

[17] Fausto N, Laird AD, Webber EM (1995). Liver regeneration. 2. Role of growth factors and cytokines in hepatic regeneration. FASEB J.

[18] McClain CJ, Barve S, Deaciuc I, Kugelmas M, Hill Det (1999). Cytokines in alcoholic liver disease. Semin Liver Dis.

[19] Matthews VB, Klinken E, Yeoh GC (2004). Direct effects of interleukin-6 on liver progenitor oval cells in culture. Wound Repair Regen.

[20] Takei R, Suzuki D, Hoshiba T, Nagaoka M, Seo SJ (2005). Role of E-cadherin molecules in spheroid formation of hepatocytes adhered on galactose-carrying polymer as an artificial asialoglycoprotein model. Biotechnol Lett.

[21] Luebke-Wheeler JL, Nedredal G, Yee L, Amiot BP, Nyberg SL (2009). E-cadherin protects primary hepatocyte spheroids from cell death by a caspase-independent mechanism. Cell Transplant.

[22] Li B, Zheng YW, Sano Y, Taniguchi H (2011). Evidence for mesenchymalepithelial transition associated with mouse hepatic stem cell differentiation. PLoS One.

[23] Katoh Y, Katoh M (2008). Hedgehog signaling, epithelial-to-mesenchymal transition and miRNA. Int J Mol Med.

[24] Hinz B, Phan SH, Thannickal VJ, Galli A, Bochaton-Piallat ML (2007). The myofibroblast, one function, multiple origins. Am J Pathol.

[25] Brenner DA, Kisseleva T, Scholten D, Paik YH, Iwaisako K (2012). Origin of myofibroblasts in liver fibrosis. Fibrogenesis Tissue Repair.

[26] Österreichera CH, Penz-Österreichera M, Grivennikovb SI, Guma M, Koltsova EK (2011). Fibroblast-specific protein 1 identifies an inflammatory subpopulation of macrophages in the liver. Proc Natl Acad Sci USA.

[27] Roskams T, Yang SQ, Koteish A, Durnez A, DeVos R (2003). Oxidative stress and oval cell accumulation in mice and humans with alcoholic and nonalcoholic fatty liver disease. Am J Pathol.

[28] Jung Y, Brown KD, Witek RP, Omenetti A, Yang L (2008). Accumulation of hedgehog-responsive progenitors parallels alcoholic liver disease severity in mice and humans. Gastroenterology.

[29] Sancho-Bru P, Altamirano J, Rodrigo-Torres D, Coll M, Millán C (2012). Liver progenitor cell markers correlate with liver damage and predict short-term mortality in patients with alcoholic hepatitis. Hepatology.

[30] Benamouzig R, Ferrière F, Guettier C, Amouroux J, Coste T (1996). Role of salivary and seric epidermal growth factor in pathogenesis of reflux esophagitis in chronic alcoholics and nondrinkers. Dig Dis Sci.

[31] Vuorela P, Sarkola T, Alfthan H, Halmesmäki E (2002). Hepatocyte growth factor, epidermal growth factor, and placenta growth factor concentrations in peripheral blood of pregnant women with alcohol abuse. Alcohol Clin Exp Res.

[32] Kömüves LG, Feren A, Jones AL, Godor E (2000). Expression of epidermal growth factor and its receptor in cirrhotic liver disease. J Histochem Cytochem.

[33] Akerman PA, Cote PM, Yang SQ, McClain C, Nelson S (1993). Long-term ethanol consumption alters the hepatic response to the regenerative effects of tumor necrosis factor-alpha. Hepatology.

[34] Jakubowski A, Ambrose C, Parr M, Lincecum JM, Wang MZ (2005). TWEAK induces liver progenitor cell proliferation. J Clin Invest.

[35] Orton RJ, Adriaens ME, Gormand A, Sturm OE, Kolch W (2009). Computational modelling of cancerous mutations in the EGFR/ERK signalling pathway. BMC Syst Biol.

[36] Jin C, Samuelson L, Cui CB, Sun Y, Gerber DA (2011). MAPK/ERK and Wnt/β-Catenin pathways are synergistically involved in proliferation of Sca-1 positive hepatic progenitor cells. Biochem Biophys Res Commun.

[37] Torii S, Yamamoto T, Tsuchiya Y, Nishida E (2006). ERK MAP kinase in G cell cycle progression and cancer. Cancer Sci.

[38] Fujiyoshi M, Ozaki M (2011). Molecular mechanisms of liver regeneration and protection for treatment of liver dysfunction and diseases. J Hepatobiliary Pancreat Sci.

[39] Yeoh GC, Ernst M, Rose-John S, Akhurst B, Payne C (2007). Opposing roles of gp130-mediated STAT-3 and ERK-1/ 2 signaling in liver progenitor cell migration and proliferation. Hepatology.

[40] Zimmers TA, McKillop IH, Pierce RH, Yoo JY, Koniaris LG (2003). Massive liver growth in mice induced by systemic interleukin 6 administration. Hepatology.

[41] Melvan JN, Siggins RW, Stanford WL, Porretta C, Nelson S (2012). Alcohol impairs the myeloid proliferative response to bacteremia in mice by inhibiting the stem cell antigen-1/ERK pathway. J Immunol.

[42] Zhang BH, Ho V, Farrell GC (2001). Specific involvement of G(alphai2) with epidermal growth factor receptor signaling in rat hepatocytes, and the inhibitory effect of chronic ethanol. Biochem Pharmacol.

[43] Sicklick JK, Choi SS, Bustamante M, McCall SJ, Pérez EH (2006). Evidence for epithelial-mesenchymal transitions in adult liver cells. Am J Physiol Gastrointest Liver Physiol.

[44] Choi SS, Diehl AM (2009). Epithelial-to-mesenchymal transitions in the liver. Hepatology.

[45] Forbes SJ, Parola M (2011). Liver fibrogenic cells. Best Pract Res Clin Gastroenterol.

[46] Choi SS, Omenetti A, Syn WK, Diehl AM (2011). The role of Hedgehog signaling in fibrogenic liver repair. Int J Biochem Cell Biol.

[47] Friedman SL (1993). Seminars in medicine of the Beth Israel Hospital, Boston. The cellular basis of hepatic fibrosis. Mechanisms and treatment strategies. N Engl J Med.

[48] Iredale JP, Benyon RC, Pickering J, McCullen M, Northrop M (1998). Mechanisms of spontaneous resolution of rat liver fibrosis. Hepatic stellate cell apoptosis and reduced hepatic expression of metalloproteinase inhibitors. J Clin Invest.

[49] Lee UE, Friedman SL (2011). Mechanisms of hepatic fibrogenesis. Best Pract Res Clin Gastroenterol.

[50] Zeisberg M, Yang C, Martino M, Duncan MB, Rieder F (2007). Fibroblasts derive from hepatocytes in liver fibrosis via epithelial to mesenchymal transition. J Biol Chem.

[51] Ueberham E, Aigner T, Ueberham U, Gebhardt R (2007). E-cadherin as a reliable cell surface marker for the identification of liver specific stem cells. J Mol Histol.

[52] Lee JM, Dedhar S, Kalluri R, Thompson EW (2006). The epithelial-mesenchymal transition: new insights in signaling, development, and disease. J Cell Biol.

[53] Barrallo-Gimeno A, Nieto MA (2005). The Snail genes as inducers of cell movement and survival: implications in development and cancer. Development.

[54] Sahlgren C, Gustafsson MV, Jin S, Poellinger L, Lendahl U (2008). Notch signaling mediates hypoxia-induced tumor cell migration and invasion. Proc Natl Acad Sci USA.

[55] Morrow D, Cullen JP, Cahill PA, Redmond EM (2008). Ethanol stimulates endothelial cell angiogenic activity via a Notch- and angiopoietin-1-dependent pathway. Cardiovasc Res.

